# Fine Mapping of a Locus Underlying the Ectopic Blade-Like Outgrowths on Leaf and Screening Its Candidate Genes in Rapeseed (*Brassica napus* L.)

**DOI:** 10.3389/fpls.2020.616844

**Published:** 2021-01-14

**Authors:** Liang Chai, Bin Feng, Xun Liu, Liangcai Jiang, Shu Yuan, Zhongwei Zhang, Haojie Li, Jinfang Zhang, Dilantha Fernando, Chun Xu, Cheng Cui, Jun Jiang, Benchuan Zheng, Lintao Wu

**Affiliations:** ^1^School of Biological Sciences, Guizhou Education University, Guiyang, China; ^2^Crop Research Institute, Sichuan Academy of Agricultural Sciences, Chengdu, China; ^3^Rape Research Institute, Guizhou Academy of Agricultural Sciences, Guiyang, China; ^4^Guizhou Oil Research Institute, Guizhou Academy of Agricultural Sciences, Guiyang, China; ^5^College of Resources, Sichuan Agricultural University, Chengdu, China; ^6^Department of Plant Science, University of Manitoba, Winnipeg, MB, Canada

**Keywords:** aberrant leaf, *Brassica napus*, *LATE MERISTEM IDENTITY1*, *REDUCED COMPLEXITY*, fine mapping, whole genome re-sequencing

## Abstract

Leaf is an important organ for higher plants, and the shape of it is one of the crucial traits of crops. In this study, we investigated a unique aberrant leaf morphology trait in a mutational rapeseed material, which displayed ectopic blade-like outgrowths on the adaxial side of leaf. The abnormal line 132000B-3 was crossed with the normal line 827-3. Based on the F_2__:__3_ family, we constructed two DNA pools (normal pool and abnormal pool) by the bulked segregant analysis (BSA) method and performed whole genome re-sequencing (WGR), obtaining the single-nucleotide polymorphism (SNP) and insertion/deletion (InDel) data. The SNP-index method was used to calculate the Δ(SNP/InDel-index), and then an association region was identified on chromosome A10 with a length of 5.5 Mbp, harboring 1048 genes totally. Subsequently, the fine mapping was conducted by using the penta-primer amplification refractory mutation system (PARMS), and the associated region was narrowed down to a 35.1-kbp segment, containing only seven genes. These seven genes were then analyzed according to their annotations and finally, BnA10g0422620 and BnA10g0422610, orthologs of *LATE MERISTEM IDENTITY1* (*LMI1*) gene from *Arabidopsis* and *REDUCED COMPLEXITY* (*RCO*) gene from its relative *Cardamine hirsuta*, respectively, were identified as the candidate genes responding to this blade-like outgrowth trait in rapeseed. This study provides a novel perspective into the leaf formation in Brassica plants.

## Introduction

Leaves are photosynthetic organs of plants, and they play a vital role in photosynthate accumulation, gas exchange, nutrient distribution, and water transport (Tsukaya, 2006; Hu et al., 2018). Leaf shape is a crucial character, and it is very important to influence the success of plants and has been refined through the evolution (Tsukaya, 2005). It can significantly affect yield and quality of crops (Zhu et al., 2016). In addition, special leaf morphology, like the functional significance of lobed leaves in rapeseed (*Brassica napus* L.), which is one of the most important oil crops globally, has been identified with potential advantages for high-density planting, which benefits mechanized harvesting in mechanical production, as well as hybrid production (Ni et al., 2017; Hu et al., 2018).

So far, it is known that leaf shape regulation is controlled by both environmental factors and genetic regulators (Tsukaya, 2005). Among the various environmental factors, the light and deficiency of nutrition can affect the leaf shape significantly. Liu et al. (2014) treated balloon flower (*Platycodon grandiflorum*) plantlets *in vitro* under different light spectra to investigate the leaf morphology and structure changes, finding that plants under monochromatic blue light treatment had larger leaf area, dry mass, and leaf thickness than those under monochromatic red light treatment or fluorescent white lamps (as control). Sotta et al. (2019) revealed that the deficiency of zinc (Zn) for *Arabidopsis* mutant *rpt5a* could lead to abnormally narrow true leaves. Radiation is another environmental factor: for example, ^12^C^6+^ heavy ion treatment caused cruciform tetraleaf conglutination and overlapped tetraleaf and twisted bileaf in *Salvia splendens* (Wu et al., 2009). In addition, atmospheric composition, like high CO_2_ level, could also change expressions of several QTLs associated with flag-leaf shape in rice (Fan et al., 2007).

As to the genetic regulators, the leaf shape-regulating network is complex, which involves many pathways and genes (Zhao et al., 2016; Xia et al., 2018). Change of some key genes would lead to abnormal leaf morphology: The *SERRATE* gene (*SE*) encodes a zinc-finger protein in *Arabidopsis*, and the *se* mutant displayed fewer leaves in rosettes and all the leaves were serrated (Prigge, 2001). Lin et al. (2019) studied the *narrow leaf1* (*nal1*) mutant of rice (*Oryza sativa* L.) representing a narrow leaf phenotype and found that *NAL1* controls leaf width through its effect on cell expansion, probably via the auxin-mediated acid growth mechanism. Moreover, even the expression of a heterologous ketohexokinase gene (*KHK*) from rat (*Rattus norvegicus*) in potato (*Solanum tuberosum* L.) could make its leaves chlorotic and crinkled, with large holes appearing between the minor veins (Geigenberger et al., 2004).

In model plant *Arabidopsis*, the *LATE MERISTEM IDENTITY1* (*LMI1*, AT5G03790) gene encodes a homeodomain leucine zipper class I (HD-Zip I) meristem identity regulator that acts together with *LEAFY* (*LFY*) to induce *CAULIFLOWER* (*CAL*) expression (Saddic et al., 2006); this gene is essential for simple serrated leaves, and function loss of it leads to bract formation (Saddic et al., 2006). In rapeseed, Ni et al. (2015, 2017) mapped an incompletely dominant locus controlling a lobed leaf trait to a 32.1-kb section on Chromosome A10 and then identified it as a *LMI1-*like gene (BnaA10g26320D) homologous to AT5G03790 in *Arabidopsis*. The overexpression of BnaA10g26320D caused serrate leaves in transgenic *Arabidopsis* (Ni et al., 2017). Moreover, Hu et al. (2018) found a *LMI1*-like gene (*BnA10.LMI1*) positively regulating the development of leaf lobes in rapeseed. Interestingly, it was also incompletely dominant and on Chromosome A10. Thus, Ni and Hu probably happened to come across the same gene. Besides, *REDUCED COMPLEXITY* (*RCO*) is a novel homeobox gene. It modulates compound leaf development by repressing growth between leaflets and thus promoting leaflet separation in *Cardamine hirsuta*, and in the *rco* mutant, leaflets are converted to lobes (Sicard et al., 2014; Vlad et al., 2014; Du et al., 2018). It exists in Brassicaceae plants such *C. hirsuta* and various *Capsella* but was evolutionarily lost in *Arabidopsis* (Vlad et al., 2014).

Unlike any of those abnormal leaf morphologies mentioned above, in this study, we report a unique aberrant leaf morphology mutant in rapeseed, which displayed ectopic blade-like outgrowths on the adaxial side of normal leaf. This blade-like outgrowth is easily recognized, even in the seedling stage, making it an efficient indicator in hybrid production. Moreover, an understanding of the molecular mechanism of it will provide a novel perspective into leaf formation and its utilization in production. It facilitates the identification of associated regions by crossing the mutant with a normal plant and then constructing the segregation population. Thus, in order to investigate the underlying genes, we constructed the F_2__:__3_ family and performed whole-genome re-sequencing (WGR) based on the two extreme DNA pools (normal pool and abnormal pool) and the two parents. Consequently, based on SNP and InDel data, one associated region was identified on chromosome A10, which was then narrowed down to a much smaller segment by the penta-primer amplification refractory mutation system (PARMS). The genes within this region were investigated by their annotations and orthologs, and then potential candidates for this blade-like outgrowth trait were screened out.

## Materials and Methods

### Plant Materials and Growing Conditions

**FIGURE 1:**
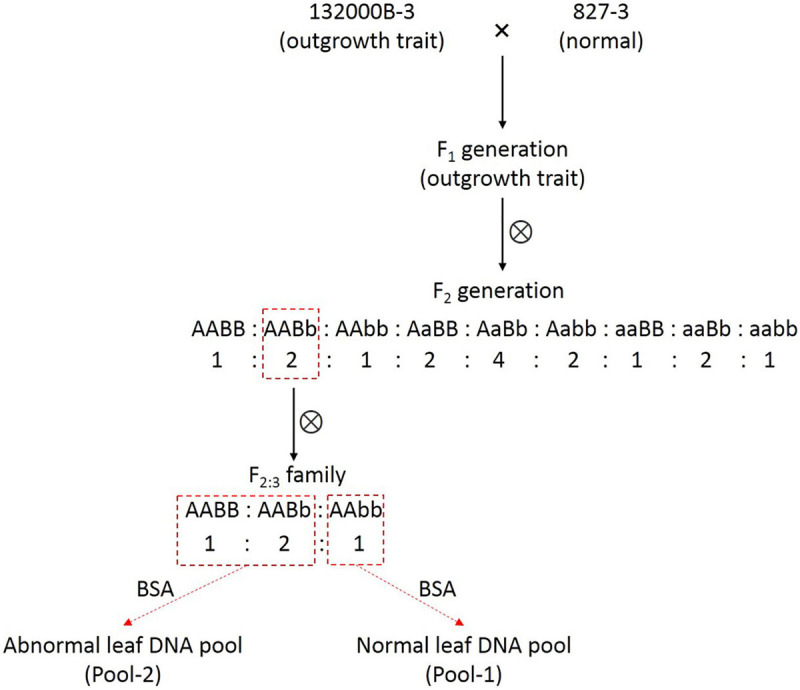
The schematic diagram for constructions of F_2:3_ family and the two DNA pools.

This blade-like outgrowth phenotype in rapeseed studied herein was originally discovered from natural mutation in the field and found controlled by two independent dominant alleles (Wei et al., 2003). The plant with abnormal leaves was then self-pollinated for several generations until the homozygous, stable line 132000B-3 was obtained. The 132000B-3 plants were then crossed with normal leaf line 827-3, which were both kept in Rape Research Institute, Guizhou Academy of Agricultural Sciences (GAAS), in order to generate the F_1_ population, followed by self-pollination of the F_1_ plants to obtain the F_2_ population. One F_2_ plant with an outgrowth trait was randomly selected and then self-pollinated to get F_2__:__3_ families (Figure 1). Then, we selected an F_2__:__3_ family which had the number of outgrowth phenotype plant: normal plants fitting the ratio of 3:1. All the plants were grown in the experimental field of GAAS under natural conditions in Kaiyang County, Guiyang, Guizhou Province.

### DNA Library Construction and High-Throughput Sequencing

Twenty normal plants and 19 plants with blade-like outgrowths in the selected F_2__:__3_ family were sampled randomly, and their DNA, as well as the DNA from parental plants 132000B-3 and 827-3, was isolated from leaves by the CTAB (cetyltrimethylammonium bromide) method (Doyle and Doyle, 1990), respectively. DNA purity was checked using the NanoPhotometer^®^ spectrophotometer (IMPLEN, CA, United States). DNA concentration was measured using Qubit^®^ DNA Assay Kit in Qubit^®^ 2.0 Fluorometer (Life Technologies, CA, United States). Equal amounts of DNA from the plants in each group were mixed to construct the two extreme pools: the normal pool (Pool-1) and the abnormal pool (Pool-2) by bulked segregant analysis (BSA) method (Michelmore et al., 1991). DNA of the two pools and the two parental plants were then utilized for paired-end sequencing library construction.

Sequencing libraries were generated using Truseq Nano DNA HT Sample preparation Kit (Illumina, United States) following the manufacturer’s recommendations, and index codes were added to attribute sequences to each sample. Briefly, the DNA sample was fragmented by sonication to a size of 350 bp, then DNA fragments were end polished, A-tailed, and ligated with the full-length adapter for Illumina sequencing with further PCR amplification. At last, PCR products were purified (AMPure XP system) and libraries were analyzed for size distribution by Agilent 2100 Bioanalyzer and quantified using real-time PCR.

These libraries constructed above were sequenced by the Illumina HiSeq 4000 platform, and 150-bp paired-end reads were generated with insert size around 350 bp.

### Filtering of Clean Reads and Alignment to the Reference Genome

To make sure reads are reliable and without artificial bias (low-quality paired reads, which mainly resulted from base-calling duplicates and adapter contamination) in the following analyses, raw data (raw reads) of fast format was firstly processed through a series of quality control (QC) procedures in-house C scripts. QC standards are as follows: (1) removing reads with ≥10% unidentified nucleotides (N); (2) removing reads with >50% bases having phred quality <5; (3) removing reads with >10 nt aligned to the adapter, allowing ≤10% mismatches; and (4) removing putative PCR duplicates generated by PCR amplification in the library construction process (read 1 and read 2 of two paired-end reads that were completely identical).

Burrows–Wheeler Aligner (BWA) (Li and Durbin, 2009) was used to align (settings: mem -t 4 -k 32 -M -R) the clean reads of each sample against the reference genome.^1^ Alignment files were converted to BAM files using SAMtools software (Li et al., 2009) (settings: -bS -t). In addition, potential PCR duplications were removed using SAMtools command “rmdup.” If multiple-read pairs have identical external coordinates, only retain the pair with the highest mapping quality.

### SNP and InDel Calling

Variant calling was performed for all samples by using the Unified Genotyper function in GATK (McKenna et al., 2010) software. SNP was used the Variant Filtration parameter in GATK (settings: –filterExpression “QD < 4.0 || FS > 60.0 || MQ < 40.0,” -G_filter “GQ < 20,” –cluster WindowSize 4). InDel was filtered by Variant Filtration parameter (settings: –filter Expression “QD < 4.0 || FS > 200.0 || Read PosRankSum < −20.0 || Inbreeding Coeff < −0.8”). ANNOVAR (Wang et al., 2010), an efficient software tool, was used to annotate SNP or InDel based on the GFF3 files for the reference genome.

### Association Region Identification and Fine Mapping of the Locus

The homozygous SNPs/InDels between two parents were extracted from the vcf files for SNP/InDel. The read depth information for homozygous SNPs/InDels above the offspring pools was gained to calculate the SNP/InDel index (Takagi et al., 2013). Δ(SNP-index) was calculated as follows (Geng et al., 2016): SNP-Index(aa) = Maa/(Paa + Maa), SNP-Index(ab) = Mab/(Pab + Mab) and Δ(SNP-Index) = SNP-Index(aa) – SNP-Index(ab). In the formulas, M and P stand for parental plants; Maa (Paa) stands for the depth of the aa group derived from M (P); and Mab (Pab) means the depth of the ab group derived from M (P). We used the genotype of one parent as the reference and the statistic read number for this parent’s genotype or the others in the offspring pool. Then, we calculated the ratio of the number of different reads in total number, which is the SNP/InDel index of the base sites. We filtered out those points whose SNP/InDel index in both pools is less than 0.3. Sliding window methods were used to present the SNP/InDel index of the whole genome. The average of all SNP/InDel indexes in each window was the SNP/InDel index for this window. Usually, we use the window size of 1 Mb and step size if 10 kb as default settings. The difference of the SNP/InDel index of the two pools was calculated as the delta SNP/InDel index.

In order to narrow down the locus, nine PARMS markers were developed (Table 1) and then used for genotyping. PARMS is a competitive allele specific PCR (KASP)-like SNP genotyping technique combining the amplification refractory mutation system (ARMS) and universal energy transfer–labeled primers (Lu et al., 2020). The markers were developed by Gentides Company, China. PARMS genotyping was performed as described in Lu et al. (2020): the thermal cycler program was (1) denaturation at 95°C for 15 min and (2) 10 cycles of denaturation at 95°C for 20 s and annealing which started at 65°C for 1 min then decreased by 0.8°C per cycle to the annealing temperature at 57°C, (3) followed by 32 cycles of denaturation at 95°C for 20 s and annealing at 57°C for 1 min. Scanning of fluorescent intensity was performed by M200 Microplate Readers (Tecan). SNP calling and plots were carried out on the SNPdecoder.^2^ Genomic DNA from 6032 individuals was isolated by the CTAB method (Doyle and Doyle, 1990).

**TABLE 1:** List of PARMS markers.

**Marker**	**SNP position**	**SNP**	**Primer sequence**
Bn30	18,554,623	C/A	P1: GAAGGTGACCAAGTTCATGCTGGGCTACAAAATTCATTATTTGAAA
			P2: GAAGGTCGGAGTCAACGGATTGGGCTACAAAATTCATTATTTGAAC
			C: GAGTTGTTGTTACTTGTAGGGCAAG
Bn35	18,703,596	T/G	P1: GAAGGTGACCAAGTTCATGCTGCGGAAGTTAACGTCGGAG
			P2: GAAGGTCGGAGTCAACGGATTGCGGAAGTTAACGTCGGAT
			C: CATCGTCGAGGAACATGAAATG
Bn65	21,210,132	C/T	P1: GAAGGTGACCAAGTTCATGCTAACATCACCACCCTCTCCTCC
			P2: GAAGGTCGGAGTCAACGGATTAACATCACCACCCTCTCCTCT
			C: GCAAGAGCTAGGCCAGGCTAG
Bn78	21,273,032	C/T	P1: GAAGGTGACCAAGTTCATGCTAAACCTCAAGGCGTTTTCGTT
			P2: GAAGGTCGGAGTCAACGGATTAAACCTCAAGGCGTTTTCGTC
			C:TTTTCCTTAGAACAACAACACGTTG
Bn81	21,295,231	G/A	P1: GAAGGTGACCAAGTTCATGCTAACTTCTTGGTAAAATTCACATCCAG
			P2: GAAGGTCGGAGTCAACGGATTAACTTCTTGGTAAAATTCACATCCAA
			C: CATTCCATTTGTAGTTTTCAATCTTTTC
Bn2132	21,326,670	A/G	P1: GAAGGTGACCAAGTTCATGCTAAATGCTATTTTATTAACTAATGCCACTA
			P2: GAAGGTCGGAGTCAACGGATTATGCTATTTTATTAACTAATGCCACTG
			C: TTGTTGGTCAGATTTTCTAAACCTC
Bn83	21,335,666	G/T	P1: GAAGGTGACCAAGTTCATGCTGGATACTTTATTCTTAGTAGTTCTAACAAATACTG
			P2: GAAGGTCGGAGTCAACGGATTGGATACTTTATTCTTAGTAGTTCTAACAAATACTT
			C: CTCACGTTAGAGTTAAAGCCATGATC
Bn2136	21,361,732	A/G	P1: GAAGGTGACCAAGTTCATGCTAAAAAGAAAAATCACAAAGAAGGTTA
			P2: GAAGGTCGGAGTCAACGGATTAAAAGAAAAATCACAAAGAAGGTTG
			C: TGCTATCAAAGTTATTCCGCTAAC
Bn84	21,376,806	G/A	P1: GAAGGTGACCAAGTTCATGCTAAGACGAGAGCGATTAGAGCTTTC
			P2: GAAGGTCGGAGTCAACGGATTAAGACGAGAGCGATTAGAGCTTTG
			C: TGTTACCCCTTACTCGACGCC

*P1: primer labeled with FAM (underlined): GAAGGTGACCAAGTTCATGCT; P2: primer labeled with HEX (underlined): GAAGGTCGGAGTCAACGGATT; C: common primer.*

### Screening and Annotation of Candidate Genes

Genes within the fine-mapped region were then aligned against the orthologs in model plant *Arabidopsis* and relative plants *Cardamine hirsute*, which had been relatively adequately studied. Moreover, the annotations of these genes were also analyzed: the Gene Ontology (GO) annotations were obtained by the Blast2GO program (Conesa et al., 2005); various pathways of the genes involved were identified by the Kyoto Encyclopedia of Genes and Genomes (KEGG) database with BLASTX and the KEGG automatic annotation server (Kanehisa et al., 2004).

## Results

### The Ectopic Blade-Like Outgrowths on the Adaxial Side of Normal Leaf Phenotype

**FIGURE 2:**
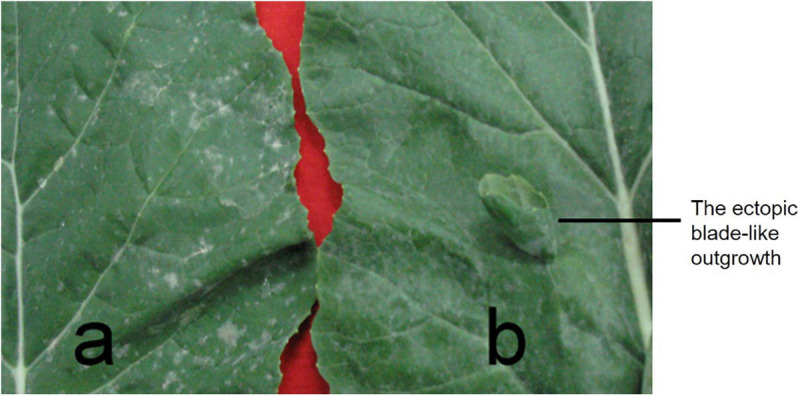
The ectopic blade-like outgrowth phenotype. **(a)** A normal leaf from rapeseed line 827-3; **(b)** the abnormal leaf from 132000B-3. One ectopic blade-like outgrowth on the adaxial side of leaf developed margins and veins, but no obvious petioles.

In 132000B-3 plants, a variable number (ranging from 1 to10) of ectopic blade-like outgrowths were discovered at the seedling stage. Their original initiation occurred on the adaxial side of the first euphylla at the five-leaf stage and is located on the side of the main vein. These outgrowths developed “leaf margins” and “veins” of themselves, but no obvious petioles, and they displayed a rolled “blade” with opposite dorsoventrality (polarity) to the leaf where they were borne (Figure 2). They grew larger as the leaf developed, and their area could reach up to 5% of the leaf. This trait happened in the first to 10th leaves from the bottom, and outgrowths on each leaf got less in upper leaves. Moreover, these blade-like outgrowths maintained until the death of the leaf. Previous research showed that this trait was controlled by two dominant alleles (Wei et al., 2003). Aiming to map one of the two alleles this time and enhance the accuracy for fine mapping it by PARMS, we screened out an F_2__:__3_ family which had the number of aberrant leaf plant: normal leaf plants fitting the expected ratio of 3:1 (Figure 1), to perform the following researches.

### Whole-Genome Re-Sequencing Data

Four DNA samples, including 132000B-3 (abnormal parent), 827-3 (normal parent), the extremely abnormal bulked DNA pool (Pool-2), and the extremely normal bulked DNA pool (Pool-1), were whole-genome re-sequenced on the Illumina HiSeq 4000 platform. A total of 93.462 Gb raw data was produced. The read number varied among the samples ranging from 14984.603 M to 31306.537 M. After filtration, 93.19 Gb clean data was left. The Q30 ratio averaged 90.135%, while the GC content averaged 37.78% (Supplementary Table 1).

Aligned with the reference genome of ZS11, the mapped rate of the four samples ranged from 98.36 to 98.58% (Supplementary Table 2). The average read depths of the parental plants and the two pools were 15.8 and 27.6, respectively, while the average numbers of mapped reads of them were approximately 105.4 M and 200.4 M, respectively (Supplementary Table 2). Thus, the quality and quantity of the data assured the success of the library construction.

Further analysis showed that when aligned to the ZS11 reference genome, the four samples generated 1848151 to 1927772 SNPs (Table 2) and 508081 to 541669 InDels (Table 3), respectively. We found 566687, 539472, 520431, and 524993 genic (including exonic and intronic) SNPs in 132000B-3, 827-3, Pool-1, and Pool-2 samples (Table 2), respectively, which theoretically resulted in 131285, 129331, 122636, and 123578 changes in amino acid sequences (including non-synonymous single-nucleotide variations, stopgain, and stoploss), accordingly. It was also found that 132000B-3, 827-3, Pool-1, and Pool-2 had 18249, 16851, 17697, and 17817 exonic (including stop gain, stop loss, frameshift deletion, frameshift insertion, non-frameshift deletion, and non-frameshift insertion) InDels (Table 3), respectively, which would alter the length of the encoded proteins. When compared to each other, the two parental lines represented 20709 genes with polymorphism.

**TABLE 2:** Numbers of different types of SNPs in each sample.

**Category**	**Sample**			
	**132000B-3**	**827-3**	**Pool-1**	**Pool-2**
Upstream	198,815	191,492	183,517	184,693
Exonic				
Stop gain	1413	1368	1369	1374
Stop loss	312	279	303	296
Synonymous	199,462	185,084	180,793	182,697
Non-synonymous	129,560	127,684	120,964	121,908
Intronic	235,940	225,057	217,002	218,718
Splicing	819	843	783	784
Downstream	169,458	164,570	157,669	158,846
Upstream/downstream	40,394	38,739	36,758	37,111
Intergenic	951,592	982,478	948,986	951,504
ts	1,101,608	1,099,459	1,057,621	1,063,042
tv	826,164	818,142	790,530	794,896
ts/tv	1.333	1.343	1.337	1.337
Total	1,927,772	1,917,601	1,848,151	1,857,938

*132000B-3, aberrant parental plant 132000B-3 displaying ectopic blade-like outgrowths on the adaxial side of leaf; 827-3, parental plant with normal leaves; Pool-1, bulked DNA pool for individuals with normal leaves from the F_2__:__3_ family; Pool-2, bulked DNA pool for individuals with ectopic blade-like outgrowths from the F_2__:__3_ family. Upstream, variant overlaps the 1-kb region upstream of the transcription start site; Exonic, variant overlaps a coding exon; Stop gain, an SNP that leads to the immediate creation of stop codon at the variant site; Stop loss, a SNP that leads to the immediate elimination of a stop codon at the variant site; Synonymous, a single-nucleotide variant that does not cause an amino acid change; Non-synonymous, a single-nucleotide variant that cause an amino acid change; Intronic, variant overlaps an intron; Splicing, variant is within 2 bp of a splicing junction; Downstream, variant overlaps the 1-kb region downstream of the transcription end site; Upstream/downstream, variant overlaps the 1-kb region upstream of transcription start site for a gene, while it overlaps the 1-kb region downstream of transcription end site of another gene at the same time; Intergenic, variant is in intergenic region; ts, transition; tv, transversion; ts/tv, the ratio of transition/transversion.*

**TABLE 3:** Numbers of different types of InDels in each sample.

**Category**	**Sample**			
	**132000B-3**	**827-3**	**Pool-1**	**Pool-2**
Upstream	88,355	82,198	85,618	86,359
Exonic				
Stop gain	311	267	302	304
Stop loss	91	84	84	85
Frameshift deletion	3690	3603	3654	3685
Frameshift insertion	3269	3123	3191	3214
Non-frameshift deletion	5712	5205	5528	5553
Non-frameshift insertion	5176	4569	4938	4976
Intronic	106,908	96,371	101,716	102,817
Splicing	1703	1622	1633	1648
Downstream	67,226	62,430	65,535	66,030
Upstream/downstream	20,893	19,013	19,687	19,947
Intergenic	237,048	228,308	241,855	242,736
Insertion	257,432	240,462	254,288	256,036
Deletion	284,237	267,619	280,737	282,605
Total	541,669	508,081	535,025	538,641

*132000B-3, aberrant parental plant 132000B-3 displaying ectopic blade-like outgrowths on the adaxial side of leaf; 827-3, parental plant with normal leaves; Pool-1, bulked DNA pool for individuals with normal leaves from the F_2:3_ family; Pool-2, bulked DNA pool for individuals with ectopic blade-like outgrowths from the F_2:3_ family. Upstream, variant overlaps the 1-kb region upstream of the transcription start site; Exonic, variant overlaps a coding exon; Stop gain, an insertion/deletion that leads to the immediate creation of stop codon at the variant site; Stop loss, an insertion/deletion that leads to the immediate elimination of a stop codon at the variant site; Frameshift deletion, a deletion causing a frameshift; Frameshift insertion, an insertion leading to a frameshift; Non-frameshift deletion, a deletion causing no frameshift; Non-frameshift insertion, an insertion leading to no frameshift; Intronic, variant overlaps an intron; Splicing, variant is within 2 bp of a splicing junction (use -splicing_threshold to change this); Downstream, variant overlaps the 1-kb region downstream of transcription end site (use -neargene to change this); Upstream/downstream, variant overlaps the 1-kb region upstream of transcription start site for a gene, while it overlaps 1-kb region downstream of transcription end site of another gene at the same time; Intergenic, variant is in the intergenic region.*

### Association Analysis and Fine Mapping

**FIGURE 3:**
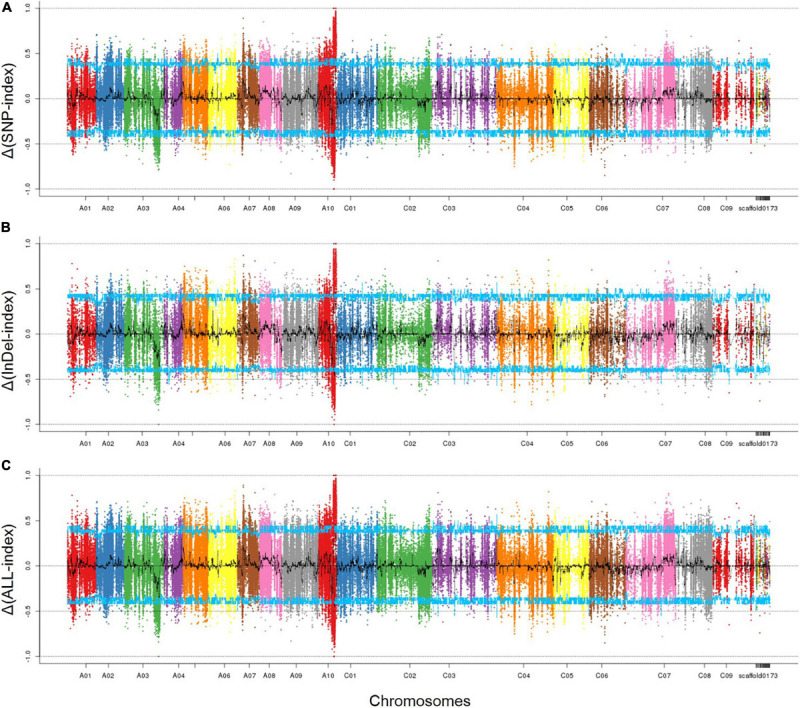
The associated region on chromosome A10 identified by SNPs and InDels. **(A)** SNP-associated region; **(B)** InDel-associated region; **(C)** overlapped associated region. The peak regions above the threshold values are defined as an associated region. The *x*-axis represents chromosomal position. The *y*-axis shows the Δ(SNP-index), Δ(InDel-index), or Δ(ALL-index), respectively. Blue line: the threshold value line for the 95% confidence intervals (*P*-value = 5%).

As described above, filtered 474,732 SNPs and 177,306 InDels were used to identify the associated region by calculating the Δ(SNP/InDel-index). Those SNPs which were homozygous in both parents, 132000B-3 and 827-3, but distinguished from each other, were kept; those with SNP/InDel–index <0.3 or read depth <7 were excluded. By combing the information of the SNP-index of Pool-1 and Pool-2, a Δ(SNP/InDel-index) was calculated and plotted against the genome positions. By examining the Δ(SNP/InDel-index) plot, peak regions above the threshold value were defined as regions where the fitted values were greater than the standard deviations above the genome-wide media. The SNP- (Figure 3A) and InDel- (Figure 3B) associated regions were highly consistent; thus, one associated region was then identified. It was in the distal end of chromosome A10, ranging from 16,388,840 to 21,907,658 bp (Figure 3C). It was 5.5 Mbp in length and contained 1048 genes.

**FIGURE 4:**
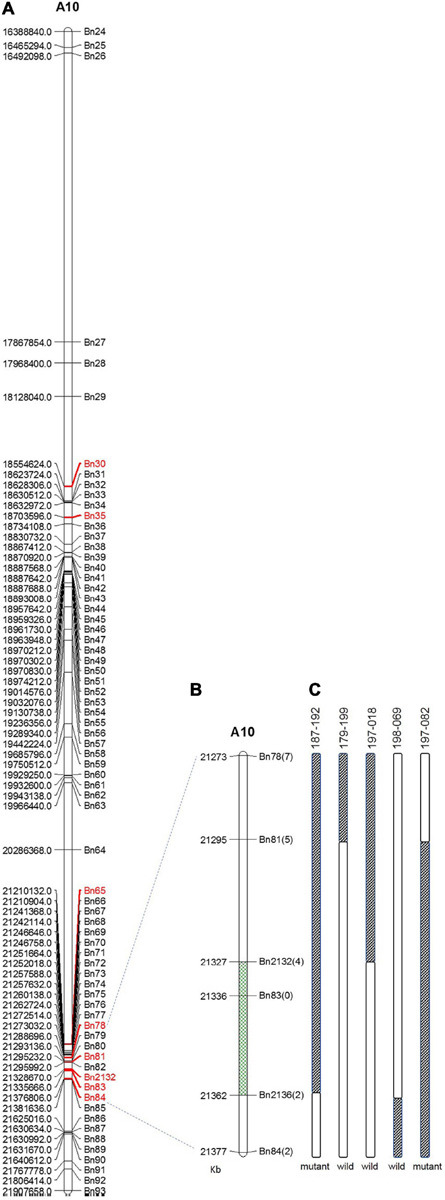
Fine mapping of the candidate gene by PARMS. **(A)** A partial physical map of PARMS markers on chromosome A10 of rapeseed; **(B)** the locus was further located between Bn2132 and Bn2136; **(C)** genotyping in recombinant plants 187–192, 179–199, 197–018, 198–069, and 197–082.

Within this region, polymorphic SNP/InDel loci between 132000B-3 and 827-3 were used to develop PARMS markers (Table 1). 6032 individual plants in the F_2__:__3_ family were tested, with randomly selected markers Bn30, Bn35, Bn65, and Bn84; consequently, 63 plants with recombinant between Bn30 and Bn84 were screened out. Comparing this with their phenotype, we located the locus in between Bn65 and Bn84. Thus, markers Bn78, Bn81, Bn2132, Bn83, and Bn2136, which were designed located in between Bn65 and Bn84, were then used to further fine-map the locus by representative recombinant plants 187–192, 179–199, 197–018, 198–069, and 197–082. Finally, this region was narrowed down to a 35.1-kb segment on chromosome A10 (Figure 4), ranging from 21,326,670 to 21,361,732 bp (from Bn2132 to Bn2136) and harboring seven genes: BnA10g0422570, BnA10g0422580, BnA10g0422590, BnA10g0422600, BnA10g0422610, BnA10g0422620, and BnA10g0422630 (Supplementary Table 3). The seven genes totally generated 125 SNPs (Supplementary Table 4) and 59 InDels (Supplementary Table 5), of which the upstream variants accounted for the largest proportion.

### Identification of Candidate Genes by Ortholog Alignment and Gene Annotations

Six of these seven genes got GO annotations, which were sorted into three categories: biological processes (BP), cellular component (CC), and molecular function (MF), which involved terms like translation (GO:0006412), structural constituent of ribosome (GO:0003735), and ribosome (GO:0005840) (Supplementary Table 6). It seemed that none of them was significantly related to the blade-like outgrowth trait, and so did the KEGG pathway (data not shown) analysis.

Thus, we had to turn to orthologs in model plant and their relatives. We aligned these genes to *Arabidopsis* and *C. hirsute*, which have been studied well and provided ortholog information in detail (Table 4): (1) BnA10g0422620 shared 81.43% amino acid sequence identity with AT5G03790 (Supplementary Figure 1A), which is identified as *LMI1* or *HOMEOBOX 51* (*HB51*) in *Arabidopsis*, encoding a HD-Zip I meristem identity regulator that is involved in floral initiation by inducing *CAL* expression together with *LFY*. It also has additional *LFY*-independent roles in leaf morphogenesis and bract formation (William et al., 2004; Saddic et al., 2006). Moreover, *LMI1* positively regulates the development of leaf lobes in rapeseed (Hu et al., 2018). (2) When aligned to *Arabidopsis* data, BnA10g0422610 also presented the highest amino acid sequence identity (60.98%) with AT5G03790, but further analysis showed that it was more homologous (68.02%) to *RCO* from *C. hirsute* (Supplementary Figure 1B). The *RCO* gene evolved in the Brassicaceae family through gene duplication and was lost in *Arabidopsis*, and the *rco* mutant of *C. hirsuta* converted its adult leaf from being dissected into a simple lobed leaf (Vlad et al., 2014).

**TABLE 4:** The seven genes on the fine-mapped locus and their orthologs in *Arabidopsis* or *C. hirsute*.

**Gene ID from *B. napus* ZS11**	**Ortholog**		
	**Species**	**Gene ID**	**Description**
BnA10g0422570	*Arabidopsis*	AT5G03850	Nucleic acid-binding, OB-fold-like protein
BnA10g0422580	*Arabidopsis*	AT5G03840	TERMINAL FLOWER 1 (TFL1)
BnA10g0422590	*Arabidopsis*	AT5G03330	Cysteine proteinase superfamily protein
BnA10g0422600	*Arabidopsis*	AT5G03795	Exostosin family protein
BnA10g0422610	*C. hirsute*	KF939591	REDUCED COMPLEXITY (RCO)
BnA10g0422620	*Arabidopsis*	AT5G03790	HOMEOBOX 51 (HB51), LATE MERISTEM IDENTITY1 (LMI1)
BnA10g0422630	*Arabidopsis*	AT5G03770	KDO transferase A (KDTA)

Of the remaining five putative genes, (3) AT5G03850 produces an oligonucleotide-binding (OB)-fold-like protein; (4) AT5G03840, identified as *TERMINAL FLOWER 1* (*TFL1*), plays an antagonistic role to FT/TSF in the determination of inflorescence meristem identity; (5) AT5G03330 encodes a cysteine proteinase superfamily protein involving protein deubiquitination or proteolysis; (6) AT5G03795, encoding an exostosin family protein, is homologous to BnA10g0422600; and (7) AT5G03770 encodes a putative KDO (3-deoxy-D-manno-octulosonate) transferase. Based on abovementioned information, BnA10g0422610 and BnA10g0422620 were considered the most likely candidate genes for this ectopic blade-like outgrowth phenotype. BnA10g0422610 harbored 44 SNPs, including 33, 9, and 2 upstream, intronic, and downstream variations, respectively (Supplementary Table 4), which would not change the sequence of its protein. Furthermore, it had eight upstream, five intronic, one exonic, and one downstream InDels (Supplementary Table 5). Notably, the exonic change was a non-frameshift insertion (CTG); it would not vary the reading frame but add one amino acid. As to BnA10g0422620, it had 5, 3, and 14 upstream, exonic, and downstream SNPs, respectively, including one non-synonymous single-nucleotide variant (SNV) in the exon (Supplementary Table 4). It also had 3, 5, and 3 upstream, intronic, and downstream InDels (Supplementary Table 5), respectively, which would not make any changes in its protein sequence. Moreover, we performed the real-time quantitative PCR (qPCR) to determine the expression of genes within the narrowed 35.1 kb region but found that BnA10g0422610 and BnA10g0422620, as well as the other seven genes, showed no change in transcriptional level (data not shown).

## Discussion

In this study, we fine-mapped a locus underlying an aberrant leaf trait, which displayed ectopic blade-like outgrowths on the adaxial side of leaf. This phenomenon has never been investigated in rapeseed, and we finally identified BnA10g0422610 and BnA10g0422620 as the potential candidate genes for this trait. Leaf morphology is precisely controlled by genetic information. To date, researches about leaf morphology mainly have focused on serrated margin (Vlad et al., 2014), lobes (He et al., 2018), width (Fujino et al., 2008), okra shape (Zhu et al., 2016), adaxial–abaxial polarity (Yuan et al., 2010; Fukushima and Hasebe, 2014), simple/compound leaf conversion (Vlad et al., 2014), *etc*., involving higher plants such as rice (*O. sativa* L., Fujino et al., 2008), *C. hirsute* (Vlad et al., 2014), cotton (*Gossypium hirsutum* and *Gossypium barbadense*, Zhu et al., 2016; He et al., 2018), kale (*Brassica oleracea* L. var. *acephala*, Ren et al., 2019), and model plant *Arabidopsis* (Yuan et al., 2010; Fukushima and Hasebe, 2014). So, investigation of abnormal leaf shape in rapeseed, an allotetraploid (AACC, 2n = 4x = 38) crop, can provide a novel understanding of the molecular mechanism from a new perspective.

Rapeseed, as a member of the Brassicaceae family and one of the most important oil crops worldwide, provides abundant resources of vegetable use, animal feed, ornamental value, and industrial erucic acid. Extensive leaf shape diversity exists in Brassica species, and rapeseed, smooth, serrated, or lobed leaf margin has been reported so far (Ni et al., 2015, 2017; Hu et al., 2018). These traits differ from each other in margin shape; however, the morphology of 132000B-3 in this study is distinguished from them: in 132000B-3, the leaf margin displays a normal shape, but some ectopic blade-like outgrowths appear unexpectedly on the adaxial side of leaf (Figure 2). The aberrant outgrowths have their blade, veins, and margin; however, they had no petiole or petiolule. Unlike those before-mentioned serrated or lobed leaves, this blade-like outgrowth trait increases the leaf area instead, which extends the blade area and confers potential capability of increasing the photosynthetic amount. Moreover, similar to the lobed-leaf character (Ni et al., 2015), this outgrowth trait can also be visualized at early stage, thus, it is supposed to distinguish the hybrids and ensure the purity of hybrid seeds too.

Since this blade-like outgrowth trait is a qualitative trait controlled by two dominant allele genes, rather than a quantitative trait controlled by multiple minor loci, there is no need to construct the recombinant inbred lines (RILs) or doubled haploid (DH) lines. Instead, the BSA method (Michelmore et al., 1991) can provide a more efficient approach to identify genes controlling extremely contrasting phenotype. The F_2_ population has been often used in this situation. However, this usually got relatively large associated regions or required a large F_2_ population with numerous individuals. In order to improve the mapping accuracy, especially the efficiency of PARMS markers, we chose to fine-map one of the two alleles for this time. Thus, our strategy is to use individuals with genotype AAxx to construct the two DNA pools; additionally, the AABb genotype plant in the F_2_ population could produce these progenies (F_2__:__3_ family), which had the number of outgrowth phenotype plant: normal plants fitting the ratio of 3:1 (Figure 1). In other words, we constructed a “one allele controlling like-population,” which had allele B segregated (BB: Bb: bb = 1:2:1) under the background of homozygous AA. Or *vice versa*, if the individuals in the F_2__:__3_ family showed the ratio of 3:1 (abnormal: normal), it can deduce that the causing plant in the F_2_ population must have the genotype AABb. Therefore, we can precisely locate one (allele B) of the two loci in this study.

So four DNA samples, including abnormal parental 132000B-3, normal parental 827-3, and the two bulked DNA pools constructed from F_2__:__3_ individuals, were then whole genome re-sequenced. The WGR has more efficient mare traditional markers such as sequence-related amplified polymorphism (SRAP) and simple sequence repeat (SSR) and generates denser SNP/InDel loci than specific-locus amplified fragment sequencing (SLAF-seq). In this study, sufficient SNP/InDel data was generated to identify the associated region.

When aligning these SNPs and InDels to reference genome, we chose the semi-winter rapeseed “ZS11” genome. In fact, there have been several rapeseed materials *de novo* sequenced, which provides and releases different reference genomes (Chalhoub et al., 2014; Bayer et al., 2017; Sun et al., 2017; Zou et al., 2019; Song et al., 2020) so far. These genomes differ from each other in sequence and even size, and that is because (1) the materials are different. For example, Darmor-*bzh* (Chalhoub et al., 2014) and Tapidor (Bayer et al., 2017) are of winter type, while ZS11 (Sun et al., 2017) is a semi-winter-type rapeseed; (2) the sequencing and assembling technologies are evolutionary. Additionally, the 827-3 plants used in this study are progenies of the “ZS11” series. Combining the above reasons, we consider ZS11 more appropriate than Darmor-*bzh* as the reference genome for this study.

The association analysis identified an associated region on chromosome A10, ranging from 16,388,840 to 21,907,658 bp. It is important to note that chromosome A10 in ZS11 is 26.6 Mbp in length (Song et al., 2020), so we can locate the associated region to that abterminal position. This region is 5.5 Mbp in length and contains 1048 genes within it. It is obviously too large for identification of candidate genes. Thus, the genotyping by PARMS was then performed. Firstly, we used four randomly chosen Bn30, Bn35, Bn65, and Bn84, which covered 2.8 Mbp length on chromosome A10. If the recombinant could be found in between Bn30 and Bn84, we would then further fine-map the locus with more markers located within this region; otherwise, if the recombinant was not found in between Bn30 and Bn84, we would then turn to new markers located out of the region “Bn30 to Bn84.” Herein, fortunately, we identified recombinant (between Bn65 and Bn84) on our first try. So we did not need to try any new markers out of the region “Bn30-Bn84”; instead, we consequently used Bn78, Bn81, Bn2132, Bn83, and Bn2136, which were located between Bn65 and Bn84 and finally narrowed down this segment to a 35.1-kb length, harboring only seven genes. This, therefore, provided the possibility of candidate gene selection.

We analyzed the GO annotations and KEGG pathways of the seven genes but unfortunately found no direct clue pointing toward this trait. So, we turned to their orthologs in *Arabidopsis*, the well-studied model plant. BnA10g0422620 is the ortholog of AT5G03790, which is identified as *LMI1* or *HB51* and encodes a HD-Zip I meristem identity regulator. Ni et al. (2015) studied the rapeseed lobed-leaf line Yuye 87 and fine-mapped the underlying gene to a 32.1-kb section of chromosome A10. Two *LMI1*-like genes, BnaA10g26320D and BnaA10g26330D, were considered the potential candidate genes. In the following research (Ni et al., 2017), they reported that the overexpression of those genes resulted in distinctly serrated leaf margins in transgenic *Arabidopsis*, indicating that BnaA10g26320D and BnaA10g26330D regulate leaf shape. Our further analysis found that the IDs BnaA10g26320D and BnaA10g26330D according to Darmor-*bzh* (Chalhoub et al., 2014), in fact, correspond to IDs BnA10g0422610 and BnA10g0422620 based on ZS11 (Sun et al., 2017); interestingly, Ni et al. (2017) also thought BnaA10g26320D (corresponding to BnA10g0422610 in this study) was the *RCO* type, while BnaA10g26330D (corresponding to BnA10g0422620) was the *LMI1* type. Hu et al. (2018) used different lobed-leaved and serrated-leaved rapeseed cultivars to investigate those two genes, finding that regulatory region polymorphisms in *LMI1* caused the expression variation and the consequently lobed leaf trait. It is indicated that *LMI1* significantly regulates leaf shape; particularly, its expression level is positively related to the lobed-leaved trait. Interestingly, in this study, BnA10g0422620 also had abundant variations in its upstream region and a non-synonymous SNV in an exon, which would probably vary its expression level, corresponding amino acid sequence, and even the protein structure.

*REDUCED COMPLEXITY* is a homeobox gene homologous to *LMI1*. In addition, it is part of a tandem gene triplication, but the *Arabidopsis* genome has only one of these genes, *LMI1* (Vlad et al., 2014). *RCO* arose from the duplication of *LMI1*-type sequences within the Brassicaceae after the divergence of *Aethionema* and before the last common ancestor of *Arabidopsis* and *Brassica* (Vlad et al., 2014). In other words, *Arabidopsis* has no *RCO*, which explains the reason why BnA10g0422620 was identified as *RCO* ortholog from *C. hirsuta* rather than *Arabidopsis*. *RCO* is necessary to *C. hirsuta* for formation of dissected leaves comprising distinct leaflets, whereas in *rco* mutant, leaflets convert to lobes. We found three SNPs and eight InDels upstream of BnA10g0422610 and one non-frameshift insertion in its exon, which potentially provides some unknown changes transcriptionally in its mRNA or even functionally in the consequent protein.

Both BnA10g0422610 and BnA10g0422620 are homeobox genes, which can always alter leaf morphology in transgenic plants (Lincoln et al., 1994). Some sorts of homeobox gene, such as *KNOTTED-LIKE HOMEOBOX* (*KNOX1*), is crucial for leaf primordium initiation; it works antagonistically against Asymmetric Leaves1/Rough Sheath 2/Phantastica (ARP) proteins to form a network (Kalve et al., 2014), determining the initiation of leaf primordium. It is noteworthy that unlike the traits mentioned in references, where the leaf morphology alters mainly in the leaf margin, this ectopic outgrowth phenotype displays a normal margin. The outgrowths appear on the adaxial side of the leaf, which should have been flat. It seems that an unexpected leaf primordium initiated at a wrong place. To the best of our knowledge, so far, there are very few reports about similar phenotypes: in the *kan1-2 kan2-1* double mutant of *Arabidopsis*, some outgrowths were found forming on the abaxial side of leaves (Eshed et al., 2004), while the *kan1 kan2 kan4* triple mutant represents ectopic leaf-like organs developing from the hypocotyl and outgrowths on the abaxial side of the cotyledons (Izhaki and Bowman, 2007). The *KANADI* (*KAN*) genes are members of the GARP transcription factor family and exhibit an embryonic expression pattern complementary to that of the Class III HD-Zip genes (Izhaki and Bowman, 2007). They are involved in lateral organ development and leaf dorsoventrality determination in *Arabidopsis* (Eshed et al., 2004), but how they caused the outgrowths on leaves remained unexplained. Interestingly, in this study, the outgrowths showed reverse dorsoventrality to the leaf that they attached on. It implies that this outgrowth phenotype is probably related to the wrong dorsoventrality establishment, of which the underlying molecular mechanism is beyond our recent recognition. In addition, as mentioned above, this outgrowth phenotype is controlled by two individual loci in fact, so it is also regulated by another locus (assigned as allele A herein). In this study, we only fine-mapped allele B and selected the candidate genes; thus, we will then probably investigate allele A by choosing another F_2__:__3_ family consisting of AABB, AaBB, and aaBB genotypes. This, as well as how alleles A and B interact with each other, needs further investigation in future.

## Conclusion

We mapped a locus for an abnormal ectopic blade-like outgrowth on leaf of rapeseed and then narrowed down this region to a 35.1 kbp length. Two homeobox genes, BnA10g0422610 and BnA10g0422620, were considered as the causal genes underlying this trait. This study enriches the understanding of the leaf formation in Brassica plants.

## Data Availability Statement

Raw data generated by WGR in this manuscript were deposited in the Sequence Read Archive (SRA) database in NCBI under accession number PRJNA658824.

## Author Contributions

LW conceived the experiment. LC and BF performed the research and wrote the manuscript. XL and CX contributed to phenotypic measurements. SY, ZZ, LJ, HL, JZ, CC, JJ, and BZ contributed to data analysis. DF interpreted data, reviewed, and revised the manuscript. All authors contributed to the article and approved the submitted version.

## Conflict of Interest

The authors declare that the research was conducted in the absence of any commercial or financial relationships that could be construed as a potential conflict of interest.

## Footnotes

1 https://www.ncbi.nlm.nih.gov/assembly/GCF_000686985.2

2 www.snpway.com

## Supplementary Material

Supplementary Figure 1 Amino acid sequence alignment of BnA10g0422610, BnA10g0422620 and their orthologs. **(A)** BnA10g0422620 shared 81.43% amino acid sequence identity with its ortholog AT5G03790 from *Arabidopsis*; **(B)** BnA10g0422610 share higher amino acid identity (68.02%) with RCO from *C. hirsute* than AT5G03790 (60.98%).

Supplementary Figure 2 Comparation of BnA10g0422610 and BnA10g0422620 between abnormal line 132000B-3 and zs11. **(A)** BnA10g0422610 in 132000B-3 (mut) had one amino acid more than that in zs11; **(B)** BnA10g0422620 in 132000B-3 (mut) changed an amino acid (T to A) compared with that in zs11.

Supplementary Table 1 Summary of the sequencing data for each sample.

Supplementary Table 2 Mapping rate and Coverage of each sample.

Supplementary Table 3 Basic information of the seven genes on the fine-mapped locus.

Supplementary Table 4 SNPs of the seven genes within the fine-mapped locus.

Supplementary Table 5 InDels of the seven genes within the fine-mapped locus.

Supplementary Table 6 GO annotation of the seven genes on the locus.
