# Supplementary figures and images for: Fine Mapping of a Locus Underlying the Ectopic Blade-Like Outgrowths on Leaf and Screening Its Candidate Genes in Rapeseed (Brassica napus L.)
Source: Front Plant Sci. 2021 Jan 14;11:616844. doi: 10.3389/fpls.2020.616844 (PMC7874103; doi:10.3389/fpls.2020.616844)

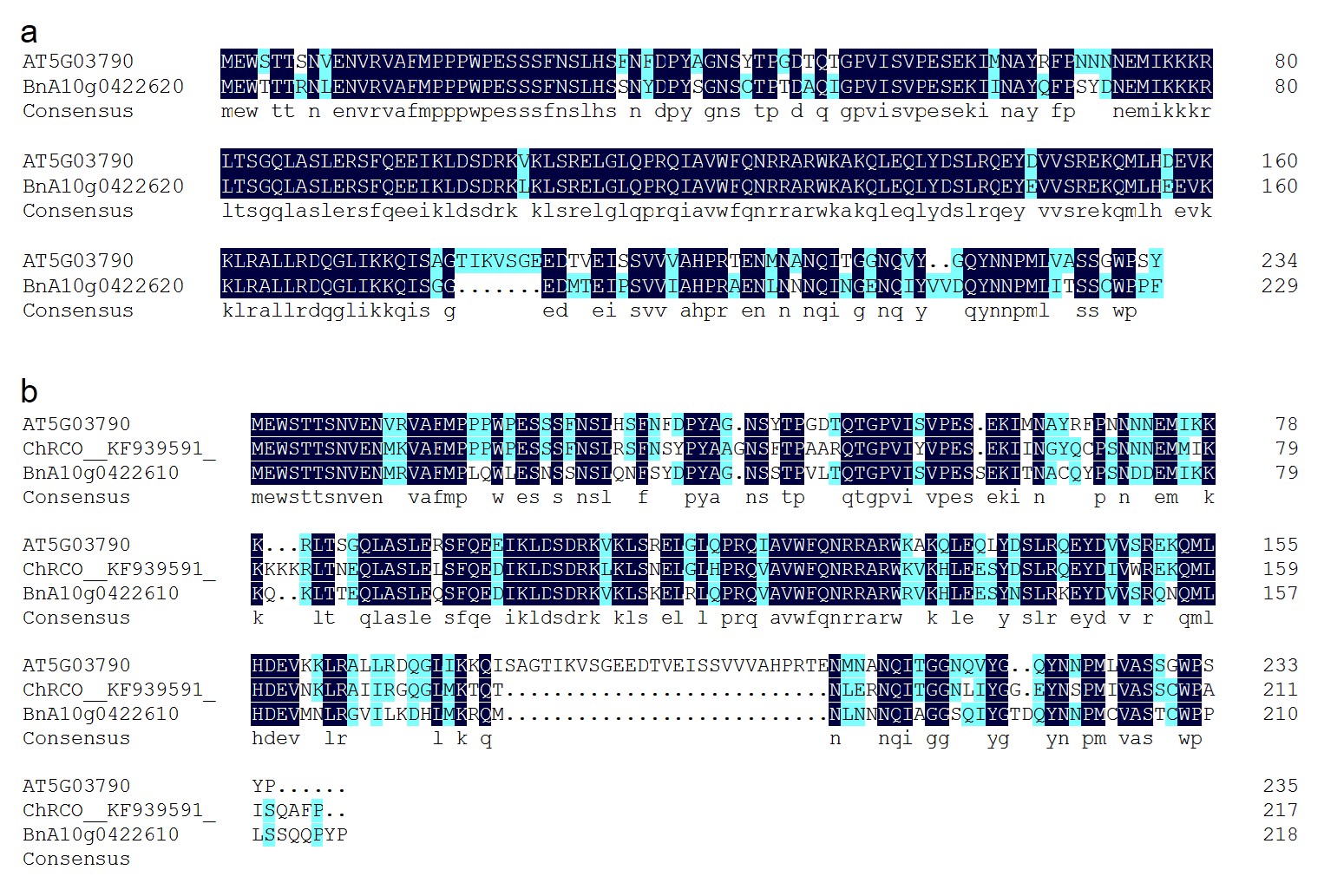

Supplement: Supplementary Figure 1 — Amino acid sequence alignment of BnA10g0422610, BnA10g0422620 and their orthologs. (A) BnA10g0422620 shared 81.43% amino acid sequence identity with its ortholog AT5G03790 from Arabidopsis; (B) BnA10g0422610 share higher amino acid identity (68.02%) with RCO from C. hirsute than AT5G03790 (60.98%). [file Image_1.JPEG]

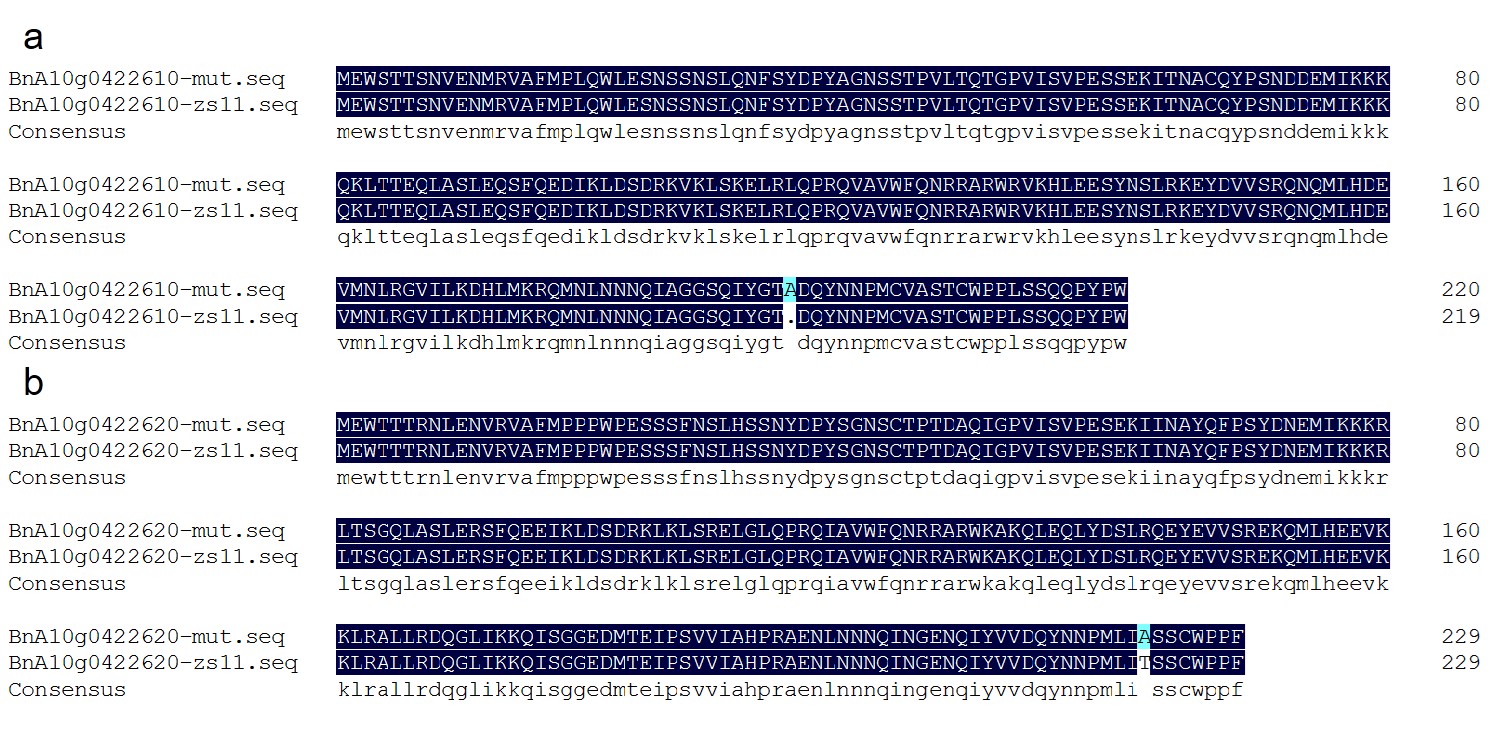

Supplement: Supplementary Figure 2 — Comparation of BnA10g0422610 and BnA10g0422620 between abnormal line 132000B-3 and zs11. (A) BnA10g0422610 in 132000B-3 (mut) had one amino acid more than that in zs11; (B) BnA10g0422620 in 132000B-3 (mut) changed an amino acid (T to A) compared with that in zs11. [file Image_2.JPEG]
