# Supplementary material for: Fine Mapping of a Locus Underlying the Ectopic Blade-Like Outgrowths on Leaf and Screening Its Candidate Genes in Rapeseed (Brassica napus L.)
Source: Front Plant Sci. 2021 Jan 14;11:616844. doi: 10.3389/fpls.2020.616844 (PMC7874103; doi:10.3389/fpls.2020.616844)
Supplement: Supplementary Table 1 — Summary of the sequencing data for each sample. [file Table_1.DOCX]

Table S1. Summary of the sequencing data for each sample

| Sample | Raw Base(bp) | Clean Base(bp) | Effective Rate(%) | Q30(%) | GC Content(%) |
| --- | --- | --- | --- | --- | --- |
| 132000B-3 | 14,984,603,100 | 14,890,396,500 | 99.37 | 93.59 | 39.35 |
| 827-3 | 17,301,945,900 | 17,267,320,500 | 99.80 | 88.92 | 37.30 |
| Pool-1 | 31,306,537,200 | 31,228,067,700 | 99.75 | 89.02 | 37.11 |
| Pool-2 | 29,868,417,900 | 29,804,242,800 | 99.79 | 89.01 | 37.36 |

Note: 132000B-3: aberrant parental plant 132000B-3 displaying ectopic blade-like outgrowths on the adaxial side of leaf; 827-3: parental plant with normal leaves; Pool-1: bulked DNA pool for individuals with normal leaves from the F_2:3_ family; Pool-2: bulked DNA pool for individuals with ectopic blade-like outgrowths from the F_2:3_ family.
