# Supplementary material for: Fine Mapping of a Locus Underlying the Ectopic Blade-Like Outgrowths on Leaf and Screening Its Candidate Genes in Rapeseed (Brassica napus L.)
Source: Front Plant Sci. 2021 Jan 14;11:616844. doi: 10.3389/fpls.2020.616844 (PMC7874103; doi:10.3389/fpls.2020.616844)
Supplement: Supplementary Table 2 — Mapping rate and Coverage of each sample. [file Table_2.DOCX]

Table S2. Mapping rate and Coverage of each sample

| Sample | Mapped reads | Total reads | Mapping rate (%) | Average depth (×) | Coverage at least 1× (%) | Coverage at least 4× (%) |
| --- | --- | --- | --- | --- | --- | --- |
| 827-3 | 113,232,169 | 115,115,470 | 98.36 | 16.65 | 93.37 | 83.48 |
| 132000B-3 | 97,649,000 | 99,269,310 | 98.37 | 14.96 | 92.39 | 81.56 |
| Pool-1 | 204,955,698 | 208,187,118 | 98.45 | 28.19 | 98.10 | 95.00 |
| Pool-2 | 195,864,831 | 198,694,952 | 98.58 | 26.95 | 98.08 | 94.91 |

Note: 132000B-3: aberrant parental plant 132000B-3 displaying ectopic blade-like outgrowths on the adaxial side of leaf; 827-3: parental plant with normal leaves; Pool-1: bulked DNA pool for individuals with normal leaves from the F_2:3_ family; Pool-2: bulked DNA pool for individuals with ectopic blade-like outgrowths from the F_2:3_ family.
