# Supplementary material for: Fine Mapping of a Locus Underlying the Ectopic Blade-Like Outgrowths on Leaf and Screening Its Candidate Genes in Rapeseed (Brassica napus L.)
Source: Front Plant Sci. 2021 Jan 14;11:616844. doi: 10.3389/fpls.2020.616844 (PMC7874103; doi:10.3389/fpls.2020.616844)
Supplement: Supplementary Table 3 — Basic information of the seven genes on the fine-mapped locus. [file Table_3.DOCX]

Table S3. Basic information of the seven genes on the fine-mapped locus

| Gene ID | Type | Start position | End position | +/- |
| --- | --- | --- | --- | --- |
| BnA10g0422570 | mRNA | 21327234 | 21327678 | + |
| BnA10g0422580 | mRNA | 21330632 | 21331697 | + |
| BnA10g0422590 | mRNA | 21332600 | 21332731 | + |
| BnA10g0422600 | mRNA | 21336508 | 21338952 | + |
| BnA10g0422610 | mRNA | 21340224 | 21341982 | - |
| BnA10g0422620 | mRNA | 21348028 | 21349414 | - |
| BnA10g0422630 | mRNA | 21358680 | 21361523 | - |
