# Supplementary material for: Fine Mapping of a Locus Underlying the Ectopic Blade-Like Outgrowths on Leaf and Screening Its Candidate Genes in Rapeseed (Brassica napus L.)
Source: Front Plant Sci. 2021 Jan 14;11:616844. doi: 10.3389/fpls.2020.616844 (PMC7874103; doi:10.3389/fpls.2020.616844)
Supplement: Supplementary Table 4 — SNPs of the seven genes within the fine-mapped locus. [file Table_4.DOCX]

Table S4. SNPs of the seven genes within the fine-mapped locus.

| Gene | SNP | Position on ChrA10 | Type | Description |
| --- | --- | --- | --- | --- |
| BnA10g0422580 | T/A | 21329722 | upstream |  |
| BnA10g0422580 | T/G | 21329993 | upstream |  |
| BnA10g0422580 | C/A | 21330957 | intronic |  |
| BnA10g0422580 | C/T | 21331661 | exonic | synonymous SNV |
| BnA10g0422590 | G/A | 21332617 | exonic | synonymous SNV |
| BnA10g0422590 | T/C | 21332637 | exonic | nonsynonymous SNV |
| BnA10g0422590 | A/G | 21332794 | downstream |  |
| BnA10g0422590 | G/A | 21332968 | downstream |  |
| BnA10g0422590 | T/G | 21332977 | downstream |  |
| BnA10g0422590 | G/T | 21333716 | downstream |  |
| BnA10g0422600 | G/T | 21335666 | upstream |  |
| BnA10g0422600 | A/T | 21336137 | upstream |  |
| BnA10g0422600 | G/A | 21336258 | upstream |  |
| BnA10g0422600 | A/C | 21337608 | intronic |  |
| BnA10g0422600 | A/G | 21338798 | exonic | synonymous SNV |
| BnA10g0422600 | T/A | 21338909 | exonic | synonymous SNV |
| BnA10g0422600 | C/T | 21338926 | downstream |  |
| BnA10g0422600 | G/A | 21338995 | downstream |  |
| BnA10g0422610 | A/T | 21340053 | downstream |  |
| BnA10g0422610 | C/A | 21340064 | downstream |  |
| BnA10g0422610 | A/G | 21340454 | intronic |  |
| BnA10g0422610 | C/A | 21340588 | intronic |  |
| BnA10g0422610 | G/C | 21340608 | intronic |  |
| BnA10g0422610 | C/T | 21340634 | intronic |  |
| BnA10g0422610 | T/C | 21340672 | intronic |  |
| BnA10g0422610 | A/C | 21341617 | intronic |  |
| BnA10g0422610 | A/G | 21341700 | intronic |  |
| BnA10g0422610 | T/A | 21341747 | intronic |  |
| BnA10g0422610 | G/C | 21341809 | intronic |  |
| BnA10g0422610 | A/T | 21342063 | upstream |  |
| BnA10g0422610 | C/G | 21342081 | upstream |  |
| BnA10g0422610 | T/A | 21342083 | upstream |  |
| BnA10g0422610 | A/T | 21342254 | upstream |  |
| BnA10g0422610 | A/T | 21342317 | upstream |  |
| BnA10g0422610 | A/T | 21342397 | upstream |  |
| BnA10g0422610 | G/T | 21342407 | upstream |  |
| BnA10g0422610 | A/T | 21342524 | upstream |  |
| BnA10g0422610 | C/T | 21342550 | upstream |  |
| BnA10g0422610 | T/A | 21342573 | upstream |  |
| BnA10g0422610 | T/A | 21342582 | upstream |  |
| BnA10g0422610 | G/A | 21342584 | upstream |  |
| BnA10g0422610 | G/A | 21342611 | upstream |  |
| BnA10g0422610 | G/A | 21342618 | upstream |  |
| BnA10g0422610 | A/G | 21342625 | upstream |  |
| BnA10g0422610 | G/A | 21342690 | upstream |  |
| BnA10g0422610 | C/T | 21342712 | upstream |  |
| BnA10g0422610 | G/A | 21342746 | upstream |  |
| BnA10g0422610 | G/A | 21342753 | upstream |  |
| BnA10g0422610 | A/C | 21342772 | upstream |  |
| BnA10g0422610 | T/C | 21342784 | upstream |  |
| BnA10g0422610 | A/G | 21342788 | upstream |  |
| BnA10g0422610 | C/T | 21342796 | upstream |  |
| BnA10g0422610 | C/T | 21342805 | upstream |  |
| BnA10g0422610 | T/G | 21342815 | upstream |  |
| BnA10g0422610 | A/G | 21342842 | upstream |  |
| BnA10g0422610 | A/G | 21342866 | upstream |  |
| BnA10g0422610 | G/T | 21342881 | upstream |  |
| BnA10g0422610 | C/A | 21342933 | upstream |  |
| BnA10g0422610 | T/C | 21342966 | upstream |  |
| BnA10g0422610 | C/T | 21342970 | upstream |  |
| BnA10g0422610 | T/C | 21342976 | upstream |  |
| BnA10g0422610 | C/T | 21342977 | upstream |  |
| BnA10g0422620 | T/C | 21347074 | downstream |  |
| BnA10g0422620 | G/T | 21347140 | downstream |  |
| BnA10g0422620 | C/T | 21347144 | downstream |  |
| BnA10g0422620 | A/G | 21347157 | downstream |  |
| BnA10g0422620 | C/T | 21347189 | downstream |  |
| BnA10g0422620 | A/G | 21347234 | downstream |  |
| BnA10g0422620 | A/C | 21347238 | downstream |  |
| BnA10g0422620 | T/C | 21347514 | downstream |  |
| BnA10g0422620 | A/G | 21347742 | downstream |  |
| BnA10g0422620 | T/C | 21347880 | downstream |  |
| BnA10g0422620 | G/A | 21347885 | downstream |  |
| BnA10g0422620 | T/G | 21347897 | downstream |  |
| BnA10g0422620 | T/G | 21347974 | downstream |  |
| BnA10g0422620 | C/A | 21348007 | downstream |  |
| BnA10g0422620 | G/A | 21348034 | exonic | synonymous SNV |
| BnA10g0422620 | T/C | 21348057 | exonic | nonsynonymous SNV |
| BnA10g0422620 | G/A | 21348413 | exonic | synonymous SNV |
| BnA10g0422620 | G/T | 21349450 | upstream |  |
| BnA10g0422620 | A/C | 21349451 | upstream |  |
| BnA10g0422620 | G/A | 21349700 | upstream |  |
| BnA10g0422620 | G/A | 21349707 | upstream |  |
| BnA10g0422620 | T/G | 21349913 | upstream |  |
| BnA10g0422630 | A/G | 21358980 | exonic | synonymous SNV |
| BnA10g0422630 | A/G | 21358999 | exonic | synonymous SNV |
| BnA10g0422630 | A/G | 21359121 | intronic |  |
| BnA10g0422630 | C/T | 21359125 | intronic |  |
| BnA10g0422630 | T/C | 21359133 | intronic |  |
| BnA10g0422630 | C/G | 21359135 | intronic |  |
| BnA10g0422630 | C/T | 21359291 | exonic | nonsynonymous SNV |
| BnA10g0422630 | T/A | 21359292 | exonic | synonymous SNV |
| BnA10g0422630 | C/T | 21359300 | exonic | nonsynonymous SNV |
| BnA10g0422630 | C/T | 21359303 | exonic | nonsynonymous SNV |
| BnA10g0422630 | G/A | 21359705 | exonic | synonymous SNV |
| BnA10g0422630 | G/T | 21360047 | intronic |  |
| BnA10g0422630 | A/G | 21360118 | exonic | synonymous SNV |
| BnA10g0422630 | T/G | 21360585 | intronic |  |
| BnA10g0422630 | A/G | 21360827 | intronic |  |
| BnA10g0422630 | A/G | 21360936 | exonic | nonsynonymous SNV |
| BnA10g0422630 | G/A | 21360953 | exonic | synonymous SNV |
| BnA10g0422630 | C/T | 21361063 | intronic |  |
| BnA10g0422630 | C/G | 21361083 | exonic | synonymous SNV |
| BnA10g0422630 | G/T | 21361101 | exonic | synonymous SNV |
| BnA10g0422630 | G/T | 21361108 | exonic | nonsynonymous SNV |
| BnA10g0422630 | G/A | 21361109 | exonic | nonsynonymous SNV |
| BnA10g0422630 | G/T | 21361150 | exonic | nonsynonymous SNV |
| BnA10g0422630 | T/C | 21361167 | exonic | synonymous SNV |
| BnA10g0422630 | C/T | 21361212 | exonic | synonymous SNV |
| BnA10g0422630 | G/C | 21361222 | exonic | nonsynonymous SNV |
| BnA10g0422630 | A/T | 21361223 | exonic | nonsynonymous SNV |
| BnA10g0422630 | G/T | 21361247 | exonic | synonymous SNV |
| BnA10g0422630 | A/G | 21361263 | exonic | synonymous SNV |
| BnA10g0422630 | C/A | 21361283 | exonic | nonsynonymous SNV |
| BnA10g0422630 | T/A | 21361306 | exonic | nonsynonymous SNV |
| BnA10g0422630 | C/G | 21361351 | upstream |  |
| BnA10g0422630 | C/T | 21361361 | upstream |  |
| BnA10g0422630 | G/A | 21361375 | upstream |  |
| BnA10g0422630 | G/A | 21361393 | upstream |  |
| BnA10g0422630 | C/T | 21361449 | upstream |  |
| BnA10g0422630 | A/C | 21361451 | upstream |  |
| BnA10g0422630 | G/C | 21361483 | upstream |  |
| BnA10g0422630 | T/C | 21361588 | upstream |  |
| BnA10g0422630 | T/A | 21361609 | upstream |  |
| BnA10g0422630 | A/G | 21361732 | upstream |  |
