# Supplementary material for: Fine Mapping of a Locus Underlying the Ectopic Blade-Like Outgrowths on Leaf and Screening Its Candidate Genes in Rapeseed (Brassica napus L.)
Source: Front Plant Sci. 2021 Jan 14;11:616844. doi: 10.3389/fpls.2020.616844 (PMC7874103; doi:10.3389/fpls.2020.616844)
Supplement: Supplementary Table 5 — InDels of the seven genes within the fine-mapped locus. [file Table_5.DOCX]

Table S5. InDels of the seven genes within the fine-mapped locus.

| Gene | InDel | Position on ChrA10 | Type | Description |
| --- | --- | --- | --- | --- |
| BnA10g0422570 | T/- | 21328246 | downstream |  |
| BnA10g0422580 | AC/- | 21329745 | upstream |  |
| BnA10g0422580 | -/A | 21329762 | upstream |  |
| BnA10g0422580 | TCT/- | 21330367 | upstream |  |
| BnA10g0422590 | -/C | 21332864 | downstream |  |
| BnA10g0422600 | AT/- | 21335954 | upstream |  |
| BnA10g0422600 | C/- | 21336217 | upstream |  |
| BnA10g0422600 | T/- | 21337771 | intronic |  |
| BnA10g0422600 | TTAT/- | 21339068 | downstream |  |
| BnA10g0422610 | AAAATCGTACAATGCGC/- | 21340111 | downstream |  |
| BnA10g0422610 | -/CTG | 21340300 | exonic | nonframeshift insertion |
| BnA10g0422610 | TTTT/- | 21340570 | intronic |  |
| BnA10g0422610 | -/AAA | 21340587 | intronic |  |
| BnA10g0422610 | -/T | 21340769 | intronic |  |
| BnA10g0422610 | -/AATAT | 21340824 | intronic |  |
| BnA10g0422610 | -/A | 21341274 | intronic |  |
| BnA10g0422610 | TAGT/- | 21342200 | upstream |  |
| BnA10g0422610 | TT/- | 21342211 | upstream |  |
| BnA10g0422610 | CTT/- | 21342492 | upstream |  |
| BnA10g0422610 | A/- | 21342512 | upstream |  |
| BnA10g0422610 | -/CAAG | 21342579 | upstream |  |
| BnA10g0422610 | -/TGATG | 21342801 | upstream |  |
| BnA10g0422610 | A/- | 21342856 | upstream |  |
| BnA10g0422610 | T/- | 21342869 | upstream |  |
| BnA10g0422620 | AAT/- | 21347106 | downstream |  |
| BnA10g0422620 | A/- | 21347115 | downstream |  |
| BnA10g0422620 | T/- | 21347420 | downstream |  |
| BnA10g0422620 | C/- | 21348817 | intronic |  |
| BnA10g0422620 | -/A | 21349042 | intronic |  |
| BnA10g0422620 | -/T | 21349073 | intronic |  |
| BnA10g0422620 | -/AT | 21349177 | intronic |  |
| BnA10g0422620 | ATT/- | 21349239 | intronic |  |
| BnA10g0422620 | -/TGTGTG | 21349659 | upstream |  |
| BnA10g0422620 | A/- | 21349750 | upstream |  |
| BnA10g0422620 | T/- | 21349849 | upstream |  |
| BnA10g0422630 | GT/- | 21358680 | downstream |  |
| BnA10g0422630 | -/A | 21358754 | downstream |  |
| BnA10g0422630 | A/- | 21358839 | downstream |  |
| BnA10g0422630 | -/A | 21359116 | intronic |  |
| BnA10g0422630 | -/G | 21359328 | intronic |  |
| BnA10g0422630 | T/- | 21359526 | intronic |  |
| BnA10g0422630 | A/- | 21359748 | intronic |  |
| BnA10g0422630 | -/A | 21360833 | intronic |  |
| BnA10g0422630 | C/- | 21360837 | intronic |  |
| BnA10g0422630 | A/- | 21360997 | intronic |  |
| BnA10g0422630 | T/- | 21361066 | intronic |  |
| BnA10g0422630 | -/C | 21361073 | intronic |  |
| BnA10g0422630 | TCTAT/- | 21361314 | exonic | frameshift deletion |
| BnA10g0422630 | -/A | 21361324 | upstream |  |
| BnA10g0422630 | -/ATACGTCGCCGT | 21361337 | upstream |  |
| BnA10g0422630 | -/T | 21361350 | upstream |  |
| BnA10g0422630 | -/T | 21361384 | upstream |  |
| BnA10g0422630 | AA/- | 21361471 | upstream |  |
| BnA10g0422630 | -/AA | 21361580 | upstream |  |
| BnA10g0422630 | A/- | 21361643 | upstream |  |
| BnA10g0422630 | AT/- | 21361651 | upstream |  |
| BnA10g0422630 | -/TGTTGCC | 21361788 | upstream |  |
| BnA10g0422630 | TAA/- | 21361949 | upstream |  |
| BnA10g0422630 | -/A | 21362102 | upstream |  |
