# Supplementary material for: Fine Mapping of a Locus Underlying the Ectopic Blade-Like Outgrowths on Leaf and Screening Its Candidate Genes in Rapeseed (Brassica napus L.)
Source: Front Plant Sci. 2021 Jan 14;11:616844. doi: 10.3389/fpls.2020.616844 (PMC7874103; doi:10.3389/fpls.2020.616844)
Supplement: Supplementary Table 6 — GO annotation of the seven genes on the locus. [file Table_6.DOCX]

Table S6. GO annotation of the seven genes on the locus

| Gene ID | GO ID | GO Term |
| --- | --- | --- |
| BnA10g0422570 | BP: GO:0006412; MF: GO:0003735; CC: GO:0005840 | BP: translation; MF: structural constituent of ribosome; CC: ribosome |
| BnA10g0422580 | BP: GO:0006623; BP: GO:0009744; BP: GO:0009910; BP: GO:0010022; BP: GO:0010228; BP: GO:0030154; BP: GO:0090344; BP: GO:1903506; MF: GO:0003712; CC: GO:0005634; CC: GO:0005773; CC: GO:0005886; CC: GO:0031982 | BP: protein targeting to vacuole; BP: response to sucrose; BP: negative regulation of flower development; BP: meristem determinacy; BP: vegetative to reproductive phase transition of meristem; BP: cell differentiation; BP: negative regulation of cell aging; BP: regulation of nucleic acid-templated transcription; MF: transcription coregulator activity; CC: nucleus; CC: vacuole; CC: plasma membrane; CC: vesicle |
| BnA10g0422590 | - | - |
| BnA10g0422600 | BP: GO:0006486; MF: GO:0016757; CC: GO:0016021 | BP: protein glycosylation; MF: transferase activity, transferring glycosyl groups; CC: integral component of membrane |
| BnA10g0422610 | BP: GO:0006355; MF: GO:0003700; MF: GO:0043565; CC: GO:0005634 | BP: regulation of transcription, DNA-templated; MF: DNA-binding transcription factor activity; MF: sequence-specific DNA binding; CC: nucleus |
| BnA10g0422620 | BP: GO:0006355; MF: GO:0043565; CC: GO:0005634 | BP: regulation of transcription, DNA-templated; MF: sequence-specific DNA binding; CC: nucleus |
| BnA10g0422630 | BP: GO:0009245; BP: GO:0036104; MF: GO:0016757; CC: GO:0005739; CC: GO:0005886 | BP: lipid A biosynthetic process; BP: Kdo2-lipid A biosynthetic process; MF: transferase activity, transferring glycosyl groups; CC: mitochondrion; CC: plasma membrane |
